# Virtual CGH: an integrative approach to predict genetic abnormalities from gene expression microarray data applied in lymphoma

**DOI:** 10.1186/1755-8794-4-32

**Published:** 2011-04-12

**Authors:** Huimin Geng, Javeed Iqbal, Wing C Chan, Hesham H Ali

**Affiliations:** 1 Department of Computer Science, University of Nebraska at Omaha, Omaha, NE 68182 USA; 2 Department of Pathology and Microbiology, University of Nebraska Medical Center, Omaha, NE 68198 USA; 3 Institute for Computational Biomedicine, Weill Medical College of Cornell University, New York, NY 10065 USA

## Abstract

**Background:**

Comparative Genomic Hybridization (CGH) is a molecular approach for detecting DNA Copy Number Alterations (CNAs) in tumor, which are among the key causes of tumorigenesis. However in the post-genomic era, most studies in cancer biology have been focusing on Gene Expression Profiling (GEP) but not CGH, and as a result, an enormous amount of GEP data had been accumulated in public databases for a wide variety of tumor types. We exploited this resource of GEP data to define possible recurrent CNAs in tumor. In addition, the CNAs identified by GEP would be more functionally relevant CNAs in the disease pathogenesis since the functional effects of CNAs can be reflected by altered gene expression.

**Methods:**

We proposed a novel computational approach, coined virtual CGH (vCGH), which employs hidden Markov models (HMMs) to predict DNA CNAs from their corresponding GEP data. vCGH was first trained on the paired GEP and CGH data generated from a sufficient number of tumor samples, and then applied to the GEP data of a new tumor sample to predict its CNAs.

**Results:**

Using cross-validation on 190 Diffuse Large B-Cell Lymphomas (DLBCL), vCGH achieved 80% sensitivity, 90% specificity and 90% accuracy for CNA prediction. The majority of the recurrent regions defined by vCGH are concordant with the experimental CGH, including gains of 1q, 2p16-p14, 3q27-q29, 6p25-p21, 7, 11q, 12 and 18q21, and losses of 6q, 8p23-p21, 9p24-p21 and 17p13 in DLBCL. In addition, vCGH predicted some recurrent functional abnormalities which were not observed in CGH, including gains of 1p, 2q and 6q and losses of 1q, 6p and 8q. Among those novel loci, 1q, 6q and 8q were significantly associated with the clinical outcomes in the DLBCL patients (p < 0.05).

**Conclusions:**

We developed a novel computational approach, vCGH, to predict genome-wide genetic abnormalities from GEP data in lymphomas. vCGH can be generally applied to other types of tumors and may significantly enhance the detection of functionally important genetic abnormalities in cancer research.

## Background

DNA Copy Number Alterations (CNAs), or chromosomal gains and losses, play an important role in regulating gene expression and constitute a key mechanism in cancer development and progression [1-3]. Comparative Genomic Hybridization (CGH) was developed as a molecular cytogenetic method for detecting and mapping such CNAs in tumor cells by comparing hybridization intensity of a tumor and a normal DNA sample [4,5]. Recently, improved resolution and sensitivity of CGH have been achieved by array CGH (aCGH) by hybridizing to arrayed genomic DNA or cDNA clones [6-9]. However, in the post-genomic era, most cancer studies have been focusing on Gene Expression Profiling (GEP) but not CGH, and as a result, a tremendous amount of GEP data have been accumulated and made publicly accessible [10-14], but few CGH studies have been performed in large series of tumor samples [15]. The enormous amount of GEP data represents an important resource for cancer research, yet it has not been fully exploited for their links to CNAs. From the literature review, most studies including GEP and CGH have been focusing on the impact of one on the other or combining the two for identifying candidate tumor suppressor genes or oncogenes [16-28]. We hypothesized that with a well-designed computational model, GEP data can be readily used to derive functionally relevant genetic abnormalities in tumor.

In this paper, we proposed a novel computational approach, virtual CGH (vCGH), to predict DNA CNAs from GEP data, which may be functionally important as impact is being evaluated at the expression level. The biological foundation for vCGH lies in the observation that a region with a chromosomal gain or loss generally results in corresponding increased or decreased mRNA expression along the aberrant loci, as reported in Diffuse Large B-Cell Lymphoma (DLBCL) [17], Mantle Cell Lymphoma (MCL) [18], Natural Killer-Cell Lymphoma (NKCL) [19], Acute Myeloid Leukemia (AML) [20], sarcoma [25], glioblastoma [27], breast cancer [21,22,28], prostate cancer [23] and gastric cancer [24]. We recently studied a large group of DLBCL and MCL samples previously GEP profiled with Lymphochip [29-31] for genetic abnormalities using CGH, and found that DNA CNAs had a substantial impact on the expression of genes in the involved chromosomal regions [17,18]. In another study on a number of tumor specimens and cell lines of NKCL using high-resolution aCGH and Affymetrix GEP microarrays, we observed a similar relationship between DNA CNAs and mRNA expression; a considerable percentage of variance in mRNA expression is directly attributable to underlying variation in gene copy numbers [19]. The association between GEP and CGH allows the development of vCGH when trained on a sufficient number of tumor samples. To our advantage, we had 190 DLBCL and 64 MCL samples examined by both CGH (Vysis CGH kits, Downers Grove, IL) and GEP (Affymetrix Inc., Santa Clara, CA). The paired GEP and CGH data on a large number of tumor samples provide a unique resource for developing and verifying the vCGH model.

vCGH was built on hidden Markov models (HMMs). HMMs are well-developed statistical models for capturing hidden patterns from observable sequential data, having been successfully applied in biology for finding CpG islands, protein secondary structure, etc. [32]. HMMs have recently been applied in aCGH for segmentation, a procedure to divide the signal ratios of each clone on the array into states, where all of the clones in a state have the same underlying copy number [33,34]. In this paper, HMM was first time used for an integrative analysis of the GEP-to-CGH prediction which intended to capture two primary sources of uncertainty embedded in genomic data: (1) the significant but subtle correlations between GEP and CGH; (2) the sequential transitions of DNA CNAs along a chromosome. Hertzberg *et al*. has developed a method for predicting chromosomal aneuploidy from GEP data using fold change and chromosomal relative expression calculation for each chromosome [35]. The major limitation with this approach is that it can only call whole chromosome gain or loss. Nilsson *et al*. proposed a method that employed total variance minimization techniques for chromosomal segmentation based on altered gene expression pattern [36]. Our proposed vCGH method differs from the previous methods in two important respects. First, the proposed vCGH is based on HMMs, which are classical pattern recognition methods with a rich set of existing estimation and inference algorithms for sequential observations. Second, the vCGH is specifically designed to train paired CGH and GEP datasets and predict CNAs using GEP data only. The special requirement of vCGH is to ensure specificity of CNA calling from the GEP data.

vCGH was aimed to enhance the limited CGH data with the wealth of GEP data and provide an integrative genomic-transcriptomic approach for identifying functionally relevant CNAs in tumor pathogenesis. Many of the common CNAs are pathogenetically significant and provide additional information on a tumor which may not be immediately evident from the CGH data. CGH in principle defines only the chromosomal structural changes, but the functional effects of CNAs can be reflected by altered gene expression. The information is important in cancer research to identify the target genes in regions of CNAs and the biological effect of the CNAs.

## Methods

In vCGH, HMMs are used to address the following question: "Given a sequence of GEP data as observations along a chromosome, predict the hidden CGH status of the chromosomal gains or losses."

### vCGH model structure

**Figure 1:**
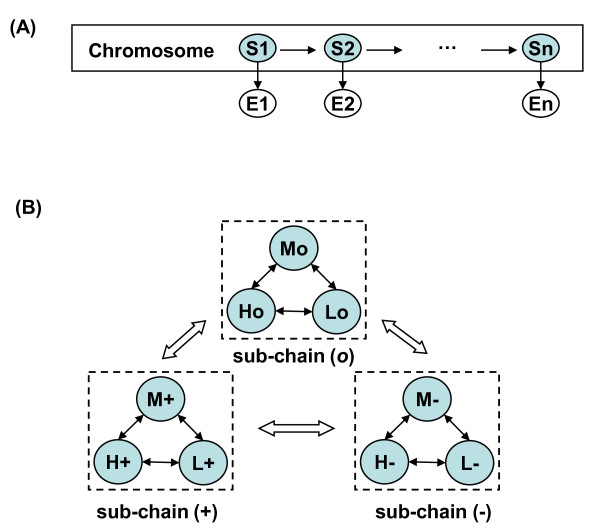
**vCGH model structure**. (A) vCGH model presented as a Bayesian network. The shaded nodes *S_1_*, *S_2_*, ..., *S_n _*represent hidden state variables for genes and the white nodes *E_1_*, *E_2_*, ..., *E_n _*represent the observations for the variables. There are three symbols for GEP observations, *"H", "L" *and *"M" *for *high*, *low *and *medium *expression, respectively. There are nine hidden states that GEP profiles superimposed on CGH, *H_+_, L_+_, M_+_, H_-_, L_-_, M_-_, H_o_, L_o _*and *M_o_*, where = *"+"*, *"-" *and "*o*" for *gain*, *loss *and *normal *CGH status, respectively. (B) State transition diagram of vCGH model. The model is a single HMM chain integrating three Markov sub-chains: (+), (-) and (*o*). In each sub-chain, a Markov chain is graphically shown as a collection of states, with arrows between them describing the state transitions within a CNA (gain, loss or normal). There are also arrows between sub-chains, describing the state transitions from one CNA to another CNA.

A HMM is a Bayesian network which describes a doubly embedded stochastic process with one observable process and one hidden process. In vCGH, the observable process {*x_i_*} describes GEP observations along a chromosome, where *x_i _="H", "L" *or *"M" *for *high*, *low *or *medium *expression of a gene; the hidden process {*π_i_*} describes the underlying CNAs, where *π_i _*= *"+"*, *"-" *or "*o*" for *gain*, *loss *or *normal *copy number status of a gene. In Figure 1A, vCGH model was illustrated as a Bayesian network, where the shaded nodes *S*_1_, *S*_2_, ..., *S_n _*represent hidden state variables and the visible nodes *E*_1_, *E*_2_, ..., *E_n _*represent observations for the variables. The emission space consists of three symbols from GEP observations {*H*, *L*, *M*} and the hidden state space consists of nine states that GEP superimposed on the CNA {*H_+_, L_+_, M_+_, H_-_, L_-_, M_- _, H_o_, L_o_, M_o_*}, where *E*_α _emits *E, E *∈ *{H, L, M} *and *α *∈ {+, -, *o*}. A hidden state *H_+_*can only emit *H*; however an emission *H *could come from any of the three underlying hidden states, *H*_+_, *H*_- _or *H*_o_. The reason that we limit the number of levels to three for GEP (*L, M, H*) and three for CGH (*-, o*, *+*) is the model complexity. Five levels for CGH (--, -, *o*, +, ++) and GEP (*LL, L, M, H, HH*) in the HMM would give 5*5 = 25 hidden states (i.e., the five GEP observations superimposed on the five CNA levels) and the transition matrix would have 25*25 = 625 parameters which is much more than the current 9*9 = 81 parameter model. Since we generally have a limited number of training samples, the three-level model is more appropriate in the current framework.

Figure 1B showed the state transition diagram of vCGH. The model is a single chain incorporating three Markov sub-chains. In each sub-chain, there is a complete set of state transitions, describing a continuous DNA segment within a gain, loss or normal CNA status. The state transitions between sub-chains are also allowed to describe the state change of a gain, loss or normal CNA. This design of intra- and inter-sub-chain transitions in vCGH makes it possible to identify alterative gain, loss and normal regions of variable length automatically.

### vCGH training and prediction

For a specific tumor type, genomic aberrations often occur in a specific set of chromosomal hotspots. For example, DLBCL has frequent aberrations involving gains of 2p, 6p and 18q and loss of 6q and 17p [17], and the hallmark aberrations of MCL are gains of 3q and 8q and losses of 1p, 6q, 8p, 9p, 9q, 11q and 13q [18]. To accurately reflect the chromosomal differences, we developed and trained a separate HMM for each chromosome so that each chromosome can have a different statistical transition and emission distributions. Our training dataset includes the paired GEP and CGH data, and hence the hidden state path for each observation sequence is known. Therefore, the transition and emission probabilities can be estimated using Maximum Likelihood Estimation (MLE) in Eq. (1) and (2),(1)(2)

where *a_kl _*is the transition probability from state *k *to state *l*, *e_l_*(*b*) is the emission probability on output symbol *b *for state *l, A_kl _*and *E_l_*(*b*) are the counts that a state transition (*k *to *l*) and that a particular emission (*b_l_*) happened in the training data. *k, l *and *l' *∈ *{H*_+_, *H*_-_, *H_o_*, *L*_+_, *L*_-_, *L_o_*, *M*_+_, *M*_-_, *M_o_*} and *b *and *b' *∈ {*H*, *L*, *M*}. The initial probabilities of the states at the beginning of the chain for each chromosome are estimated using MLE, *pi*(*l*) = *N_l_/N*, where *pi*(*l*) is the initial probability for state *l*, *N_l _*is the number of samples with state *l *at the beginning of the chain, and *N *is the total number of samples in the training data.

Having the vCGH parameters trained by the paired GEP and CGH data in the training dataset, we used Viterbi and Posterior (also called *Forward *and *Backward*) decoding algorithms [32] to predict hidden CGH states based on the GEP observations for a new tumor sample in the testing dataset. Viterbi algorithm works by finding the highest probability path as a hidden state path, whereas Posterior algorithm finds the most likely state for each position and then concatenate those states into a hidden state path. The detailed algorithms of Viterbi and Posterior were given in Additional file 1. Preliminary versions of vCGH Viterbi and vCGH Posterior methods were presented in conferences by Geng *et al*. [37-39].

An alternative inference method for HMM when given only emissions as training data, i.e., only GEP observations in training, is the Baum-Welch algorithm [32]. Baum-Welch algorithm estimates the model parameters (transition and emission probabilities) together with unknown CGH states by an iterative procedure. We chose not to use this algorithm, as there are many parameters in the model but relatively few data points at each gene position to estimate these parameters. Instead, we used the Viterbi or Posterior algorithms in which the true CGH states were used to guide the HMM prediction.

### vCGH validation

**Figure 2:**
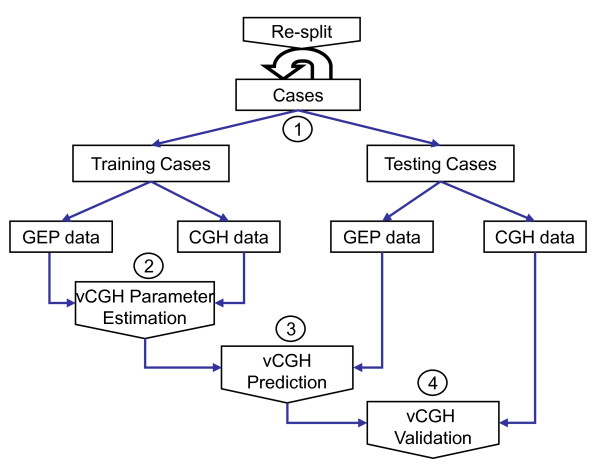
**Procedure of vCGH training and validation**. The entire dataset was split into training and testing datasets. In the training dataset, the paired GEP and CGH data were used for model parameter estimation; in the testing dataset, only the GEP data was used for vCGH prediction and the corresponding CGH data was used for validating vCGH prediction. The whole process was repeated by different splitting of training and testing datasets and the model performance was evaluated by LOOCV.

The procedure of vCGH was illustrated in Figure 2. The entire dataset was split into training and testing datasets. In the training dataset, the paired GEP and CGH data were used for HMM parameter estimation, and in the testing dataset, only the GEP data of a tumor sample was used to predict the CNAs. The predicted gain, loss or normal status of each gene was compared with those from the experimental CGH on the same tumor samples using the criteria of sensitivity, specificity and accuracy to validate vCGH. The entire process was repeated and the model performance was evaluated by Leave-One-Out Cross Validation (LOOCV). The sensitivity, specificity and accuracy can be calculated from the 2 × 2 contingency table for gain and loss. For example, in the contingency table for gain, true positive (TP) is the number of genes as a gain by both CGH and vCGH, true negative (TN) is the number of genes not as a gain by both CGH and vCGH, false positive (FP) is the number of genes as a gain by vCGH but not by CGH, and false negative (FN) is the number of genes as a gain by CGH but not by vCGH. Then, Sensitivity = TP/(TP+FN), Specificity = TN/(TN+FP), and Accuracy = (TP+TN)/(TP+TN+FP+FN). The same statistics were calculated for loss as well.

We also created two other methods to compare with vCGH, named rGEP (raw GEP) and sGEP (smoothing GEP), by simply mapping GEP status to CGH status without an intelligent learning and predicting process. By rGEP, we mean that a high expression status of a gene is mapped to a gain ("*H*" → "*+*"), low expression mapped to loss ("*L*" → "-"), and medium expression mapped to normal ("*M*" → "*o*"). In sGEP, a smoothing method (a multinomial model, as described below) was applied after rGEP to get a gain or loss status for a chromosomal cytoband, which contains a number of consecutive genes.

### Smoothing algorithm

Since gains and losses identified by our experimental CGH reflected the resolution in cytobands, we determined as well the gains and losses on cytoband resolution for vCGH by applying a smoothing method. Basically, a multinomial probability was used to measure the likelihood of a cytoband harboring a gain or loss. In Eq. (3), *L *is the likelihood under a hypothesis *H*, where *H*_0 _is the null hypothesis that "a cytoband is not harboring a gain or loss" and *H*_1 _is the alternative hypothesis that "a cytoband is harboring a gain or loss"; *n*_+_, *n*_- _and *n*_o _are the numbers of genes in the gain, loss or normal status, and *n *is the total number of genes on this cytoband (*n *= *n*_+_+*n*_-_+*n*_o_); *θ*_+_, *θ*_- _and *θ*_o _are the corresponding multinomial parameters which can be estimated using MLE in Eq.(4). Under *H*_1 _hypothesis, *θ*_1,+_, *θ*_1,- _and *θ*_1, o _are estimated by the number of genes *n*_+_, *n*_- _and *n*_o _on a cytoband; Under *H*_0 _hypothesis, *θ*_0,+_, *θ*_0,- _and *θ*_0, o _are estimated by the number of genes *N*_+_, *N*_- _and *N*_o _on the whole genome as the background (*N *= *N*_+_+*N*_-_+*N*_o_). Log-of-odds (LOD), which is *Log*10 of the ratio of the two likelihoods, was used to measure the likelihood that a cytoband harbors a gain or loss, as described in Eq.(5). The higher the LOD score, the more likely a cytoband harbors a genomic gain or loss.(3)(4)(5)

### Sample description and data processing

The GEP and CGH experiments were performed on 190 DLBCLs [17] and 64 MCLs [18]. The survival data was also available for 190 DLBCL patients, who were all treated with CHOP (a regimen of cyclophosphamide, doxorubicin, vincristine and prednisone). The GEP data were obtained using Affymetrix HG-U133 plus2 arrays and normalized (global median normalization) using BRB-Array Tool [40]. The gene expression values (continuous variable) were discretized into three distinct levels, *"H", "L" *or *"M*", representing high, low or medium gene expression, respectively. 1.5-fold change was used as the threshold to determine high (>1.5fold increase), low (>1.5fold decrease) or medium (between 1.5 fold increase and decrease) expression of a gene in a tumor as compared to the median expression of the gene across the tumor cohort. The CGH experiments were performed by Vysis CGH kits (Downers Grove, IL). aCGH-Smooth [41] was used to determine breakpoints and relative levels of DNA copy number. The company recommended 1.25 and 0.75 signal ratio of tumor to normal cells was used to segregate gain (>1.25), loss (<0.75) and normal (between 0.75 and 1.25) chromosomal regions. Small-sized chromosomes and sex chromosomes were excluded from the study due to technical limitation and lack of gender data, including chromosomes 19-22, X and Y.

For a gene on GEP, we actually refer to the probeset level data without averaging multiple probesets within the same gene. A probeset in GEP data would be marked with "+" or "-" if its chromosomal locations were covered by the start and the end of a gain or a loss region from the CGH data; Otherwise it was marked with "o" representing not covered by a gain or loss region. The chromosomal locations of probesets, genes and cytobands were obtained by Affymetrix probesets alignments and NCBI Human Genome database Build 36.1. The vCGH model is based on HMMs that consider expression probesets as a sequence of hidden states without considering the distance between probesets. The vast majority of the expression probesets were near the 3' end of coding region and probesets located at other regions were equally treated. The LOD score of 2 was used as the cutoff to call a gain or loss for a cytoband after the smoothing algorithm.

### Association of gene expression and survival time with recurrent abnormalities

In order to determine whether the additional recurrent abnormalities identified by vCGH are associated with altered gene expression or not, we performed a permutation test as follows. 1) Consider all probesets (genes) that are in the region of a recurrent abnormality. 2) For each probeset calculate a one-sided Student's t-test p-value for the difference in gene expression between the samples that exhibit the recurrent abnormality, and those that are wild type for that abnormality, in the direction of increased gene expression being associated with increased copy number or decreased gene expression being associated with decreased copy number. 3) Generate a statistic equal to the sum of the log (p-values) for the genes in the region. 4) Randomly permute sample labels as gain, loss or normal according to the abnormality and repeat steps "1-3" 1000 times. 5) Calculate how many times the unpermuted statistic is smaller than the same statistics calculated with the permuted data. For example, the significance of a recurrent abnormality associated with the gene expression in this region is 0.05 if 95% of the time the sum of log (p-value) for the real data is less than that of the permuted data.

In order to determine whether the additional recurrent abnormalities identified by vCGH were associated with survival time or not, we performed survival analysis on the patient groups defined by the recurrent abnormality. Overall survival (OS) distributions were estimated using the Kaplan-Meier method and the patient groups were compared with the log-rank test.

The vCGH source code and the GEP and CGH data for DLBCL and MCL can be accessed at: http://vcgh.sourceforge.net.

## Results and discussion

Using cross-validation, vCGH was applied to 190 DLBCLs and 64 MCLs on which both GEP and CGH data were available [17,18]. vCGH was first trained by the paired GEP and CGH data on the same tumor samples in the training dataset, and then applied to the GEP data of a new tumor sample in the testing dataset to predict its CNAs. The predicted gains and losses were compared with those identified by experimental CGH on both on gene level and cytoband level.

### Gene-level validation of vCGH

**Figure 3:**
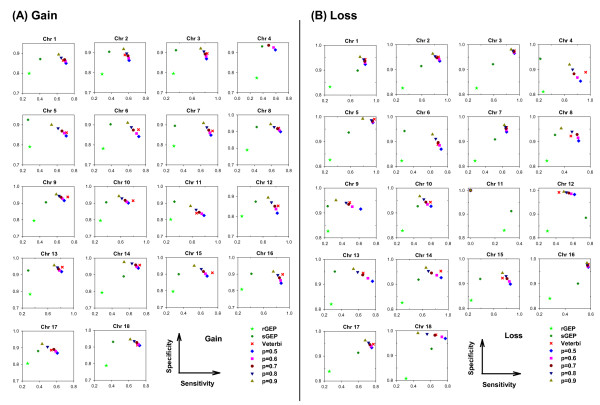
**Sensitivity and specificity of vCGH, rGEP and sGEP compared with CGH on 190 DLBCLs**. (A) Gain. (B) Loss. Each subfigure represents one chromosome. Sensitivity and specificity was shown on X and Y axis, respectively. Different prediction methods were shown in colors-- rGEP in light green, sGEP in dark green, vCGH Viterbi in red, and vCGH Posterior in a series of colors representing different posterior probability cut-offs (p = 0.5, 0.6, 0.7, 0.8 and 0.9).

**Figure 4:**
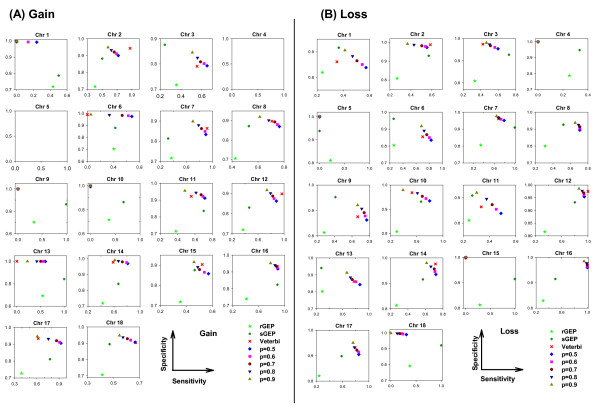
**Sensitivity and specificity of vCGH, rGEP and sGEP compared with CGH on 64 MCLs**. (A) Gain. (B) Loss. Each subfigure represents one chromosome. Sensitivity and specificity was shown on X and Y axis, respectively. Different prediction methods were shown in colors-- rGEP in light green, sGEP in dark green, vCGH Viterbi in red, and vCGH Posterior in a series of colors representing different posterior probability cut-offs (p = 0.5, 0.6, 0.7, 0.8 and 0.9).

We first evaluated vCGH, and for comparison purpose rGEP and sGEP as well, using sensitivity, specificity and accuracy against experimental CGH, in predicting gains and losses for all the DLBCL or MCL samples using LOOCV. Tables 1 and 2 summarized the sensitivity, specificity and accuracy for all chromosomes on DLBCL and MCL datasets, respectively. Figures 3 and 4 showed the performance on individual chromosomes for DLBCL and MCL datasets, respectively.

**Table 1:** Sensitivity, specificity and accuracy of vCGH*, rGEP and sGEP on DLBCL dataset

		Sensitivity (%)	Specificity (%)	Accuracy (%)
	rGEP	29.22 ± 2.34	79.36 ± 0.86	76.69 ± 1.65
	
**Gain**	sGEP	38.50 ± 6.91	90.52 ± 1.86*	87.72 ± 2.30
	
	vCGH	**69.63 ± 15.76**	89.91 ± 3.53	**89.05 ± 3.64**

	rGEP	27.82 ± 2.30	82.62 ± 0.81	81.54 ± 1.70
	
**Loss**	sGEP	47.43 ± 15.96	92.07 ± 1.69	90.90 ± 1.78
	
	vCGH	**67.27 ± 23.56**	**95.13 ± 3.23**	**94.73 ± 3.52**

*Viterbi method was used to represent vCGH.

**Table 2:** Sensitivity, specificity and accuracy of vCGH*, rGEP and sGEP on MCL dataset

		Sensitivity (%)	Specificity (%)	Accuracy (%)
	rGEP	38.45 ± 2.93	71.43 ± 0.58	69.46 ± 1.50
	
**Gain**	sGEP	42.77 ± 10.42	86.33 ± 2.97	83.59 ± 3.48
	
	vCGH	**74.49 ± 17.77**	**88.56 ± 4.78**	**87.50 ± 5.59**

	rGEP	28.26 ± 3.70	80.94 ± 0.70	77.71 ± 3.47
	
**Loss**	sGEP	50.66 ± 24.13	92.71 ± 2.00*	89.19 ± 3.63
	
	vCGH	**59.63 ± 17.22**	90.63 ± 4.91	**89.30 ± 5.69**

*Viterbi method was used to represent vCGH.

On the DLBCL dataset, in Figure 3, each box represents one chromosome. Good predictions should be at the upper right corner, where both sensitivity and specificity are good; while poor predictions are the points at the lower left corner. It is obvious from Figure 3 that vCGH, both Viterbi (in red) and Posterior (in multiple colors representing different posterior probability cut-offs) methods, predict better than rGEP (in light green) and sGEP (in dark green) by lying at the most upper right corner. On most of the chromosomes, vCGH achieved 70-80% sensitivity and 90%-95% specificity, for both gain and loss prediction; while sensitivity was much lower in rGEP (30%) and sGEP (40%-50%), and specificity was also lower in rGEP (80%) and sGEP (90%). We also observed that vCGH Viterbi and vCGH Posterior had similar performance (Viterbi point lied among a series of Posterior points), and that as expected, in vCGH Posterior, specificity increases and sensitivity decreases as the posterior probability cut-off increases. The results on the MCL dataset were similar as in DLBCL dataset (Figure 4). On average, vCGH achieved 75% sensitivity and 90% specificity for gain, and 60% sensitivity and 90% specificity for loss, while sensitivity was 40% for gain and 30% for loss in rGEP, and 40% for gain and 50% for loss in sGEP, and specificity was 70% for gain and 80% for loss in rGEP, and 85% for gain and 90% for loss in sGEP. In Tables 1 and 2, performance of vCGH, rGEP and sGEP were summarized. The bold-highlighted were the best predictions, which all fell into the vCGH category except one where sGEP is marginally better than vCGH. Tables S1 and S2 in Additional file 2 showed the detailed sensitivity, specificity and accuracy of vCGH on each chromosome.

Those results suggested that vCGH was able to capture the hidden genomic CNA information buried in the GEP data, while rGEP and sGEP didn't work well, which directly map GEP status to CGH status without any learning process. We noticed that vCGH did not predict well on some chromosomes, such as gain on chromosome 4 and loss on chromosome 11 for DLBCL (Figure 3) and gain on chromosomes 1, 6, 9, 10 and 13 and loss on chromosomes 4, 5, 15 and 18 for MCL (Figure 4). This is due to infrequent aberrations and hence insufficient training data for the gains or losses on those chromosomes. For example, in 190 DLBCLs, the number of samples with chr4 gain is n = 7 and with chr11 loss is n = 1; in 64 MCLs, the number of samples with gains on chr1 is (n = 1), chr6 (n = 3), chr9 (n = 1), chr10 (n = 2) and chr13 (n = 1), and with losses on chr4 (n = 2), chr5 (n = 1), chr15 (n = 1) and chr18 (n = 2).

### Cytoband-level validation of vCGH

Cytobands are defined as the chromosomal areas distinguishable from other segments by appearing darker or lighter by one or more banding techniques for karyotype description. Our experimental CGH detected chromosomal gains and losses on cytobands. To compare vCGH with experimental CGH on the same resolution, we also determined the gains and losses on cytobands by applying a smoothing algorithm in vCGH as described in Method section.

**Figure 5:**
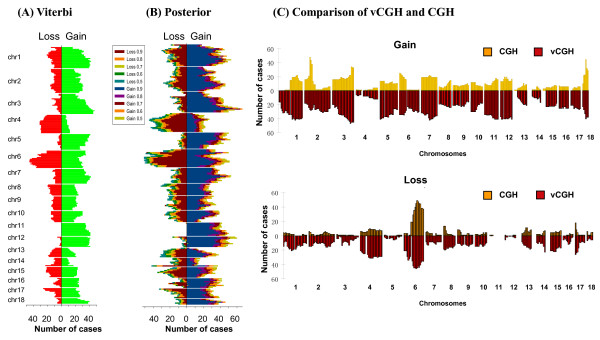
**Cytoband gains and losses by vCGH and CGH on 190 DLBCLs**. (A) vCGH Viterbi. (B) vCGH Posterior with a series of posterior probability cut-offs (p = 0.5, 0.6, 0.7, 0.8 and 0.9). (C) Comparison of vCGH and CGH where Viterbi method was used to represent vCGH. In (A) and (B), losses were shown on left-sided bars and gains were shown on right-sided bars. On Y axis are the cytobands ordered from pter to qter for each chromosome (from top to bottom). On X axis are the gain and loss frequencies, i.e. the number of samples harboring a gain or loss on a cytoband. In (C), gain and loss were shown separately in the top and bottom panels. In each panel, CGH results were shown in yellow (above X axis) and vCGH prediction were shown in red (below X axis). On X axis are the cytobands ordered from pter to qter, from chr1 to chr18. On Y axis, the height of each bar indicates gain or loss frequencies, i.e., the number of samples harboring a gain or loss on a cytoband.

**Figure 6:**
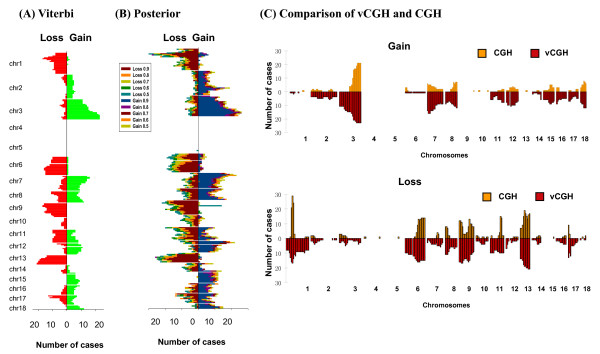
**Cytoband gains and losses by vCGH and CGH on 64 MCLs**. (A) vCGH Viterbi. (B) vCGH Posterior with a series of posterior probability cut-offs (p = 0.5, 0.6, 0.7, 0.8 and 0.9). (C) Comparison of vCGH and CGH where Viterbi method was used to represent vCGH. In (A) and (B), losses were shown on left-sided bars and gains were shown on right-sided bars. On Y axis are the cytobands ordered from pter to qter for each chromosome (from top to bottom). On X axis are the gain and loss frequencies, i.e. the number of samples harboring a gain or loss on a cytoband. In (C), gain and loss were shown separately in the top and bottom panels. In each panel, CGH results were shown in yellow (above X axis) and vCGH prediction were shown in red (below X axis). On X axis are the cytobands ordered from pter to qter, from chr1 to chr18. On Y axis, the height of each bar indicates gain or loss frequencies, i.e., the number of samples harboring a gain or loss on a cytoband.

Figures 5 and 6 showed the results of cytoband level gains and losses on DLBCL and MCL, respectively. The two vCGH decoding methods, Viterbi and Posterior, were shown in panels A and B, respectively. In each panel, loss frequencies were shown on left-sided bars and gain frequencies on right-sided bars. We found in Posterior decoding, as expected, the frequencies of gains and losses decrease as posterior probability increases (p = 0.5, 0.6, 0.7, 0.8 and 0.9) (panel B in Figures 5 and 6), and the frequencies at different posterior probability cut-offs are highly correlated, with Pearson's correlation coefficients around 0.99 (Tables 3 and 4). Comparing the results from Viterbi (panel A in Figures 5 and 6) and Posterior (panel B in Figures 5 and 6), a high concordance was also observed with Pearson's correlation coefficients around 0.95 (Tables 3 and 4). In panel C (Figures 5 and 6), the Viterbi method was used to represent vCGH to compare with the experimental CGH side by side. Gains and losses were shown separately. CGH results were above the X-axis in yellow and vCGH results were below the X-axis in red. Apparently, the majority of the recurrent gains and losses predicted by vCGH are in good concordance with those identified by experimental CGH, such as gains of 1q, 2p16-p14, 3q27-q29, 6p25-p21, 7, 11q, 12 and 18q21 and losses of 6q, 8p23-p21, 9p24-p21 and 17p13 on DLBCL. The Pearson's correlation coefficients between vCGH and CGH are around 0.8 for gains and losses (Tables 3 and 4).

**Table 3:** Correlation of gain and loss frequencies* on cytobands among CGH, vCGH Viterbi and vCGH Posterior on DLBCL dataset

	CGH	vCGH Viterbi	vCGH Posterior				
			**p = 0.5**	**p = 0.6**	**p = 0.7**	**p = 0.8**	**p = 0.9**

**CGH**	1	0.860	0.804	0.811	0.821	0.830	0.843

**vCGH Viterbi**	0.777	1	0.975	0.974	0.974	0.971	0.964

**vCGH Posterior **p = 0.5	0.653	0.941	1	0.996	0.991	0.985	0.975

p = 0.6	0.671	0.945	0.994	1	0.996	0.99	0.98

p = 0.7	0.683	0.948	0.987	0.993	1	0.995	0.987

p = 0.8	0.696	0.951	0.980	0.988	0.993	1	0.992

p = 0.9	0.713	0.943	0.966	0.975	0.981	0.988	1

*Gain was shown in the bottom left triangles, and loss in the top right triangles.

**Table 4:** Correlation of gain and loss frequencies* on cytobands among CGH, vCGH Viterbi and vCGH Posterior on MCL dataset

	CGH	vCGH Viterbi	vCGH Posterior				
			**p = 0.5**	**p = 0.6**	**p = 0.7**	**p = 0.8**	**p = 0.9**

**CGH**	1	0.766	0.734	0.745	0.744	0.752	0.756

**vCGH Viterbi**	0.828	1	0.970	0.978	0.978	0.978	0.973

**vCGH Posterior **p = 0.5	0.831	0.978	1	0.990	0.986	0.982	0.969

p = 0.6	0.828	0.980	0.996	1	0.996	0.991	0.978

p = 0.7	0.828	0.983	0.992	0.995	1	0.993	0.981

p = 0.8	0.827	0.985	0.988	0.992	0.996	1	0.990

p = 0.9	0.820	0.981	0.983	0.986	0.991	0.993	1

*Gain was shown in the bottom left triangles, and loss in the top right triangles.

As described in the model design in the Methods section, with intra- and inter- Markov sub-chain transitions, vCGH can identify alterative gain, loss or normal DNA segments automatically. vCGH is basically a segment-level prediction tool, and genes within a segment can be considered as the unit of a segment. Sensitivity, specificity and accuracy of vCGH on gene level and on cytoband level were compared in Tables S3 and S4 (Additional file 2) for DLBCL and MCL, respectively. As expected, the gene-level and cytoband-level vCGH gave very similar prediction sensitivity, specificity and accuracy.

### Additional recurrent gains and losses predicted by vCGH

**Figure 7:**
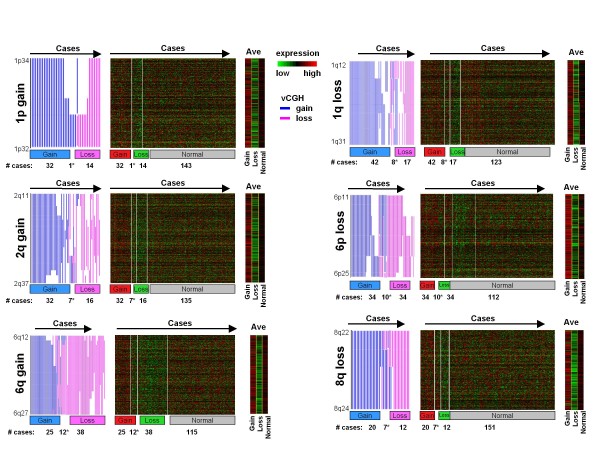
**Additional recurrent gains and losses predicted by vCGH in 190 DLBCLs**. The recurrent gains of 1p, 2q and 6q and losses of 1q, 6p and 8q were shown. For each of those regions, DLBCL samples were split into gain, loss or normal groups according to the vCGH prediction. The gains and losses were displayed on the left side of each panel, with each vertical line representing a tumor sample (gain in pink and loss in blue). The terminals of each line represent the start and the end of a gain or loss. Some samples harbored both gains and losses in the region and were displayed in the middle between the gain and loss groups (marked by *). The expression of the genes in the region was shown in heatmap on the right side of each panel. The samples were ordered the same way in the heatmap as in the gains and losses on the left side. The number of samples in each group was listed under the heatmap. The average gene expression in each group (gain, loss or normal) was also plotted.

In addition to the common recurrent gains and losses between vCGH and CGH, vCGH also predicted some recurrent gains or losses that were not observed in CGH, such as gains of 1p (in 33 out of 190 samples), 2q (39/190) and 6q (37/190) and losses of 1q (25/190), 6p (44/190) and 8q (19/190) on the DLBCL dataset (Figure 5C). We checked those additional recurrent abnormalities predicted by vCGH and the corresponding gene expression within those regions in Figure 7. We observed higher expression of genes for the gain region and lower expression of genes for the loss region, as compared to the normal group.

**Figure 8:**
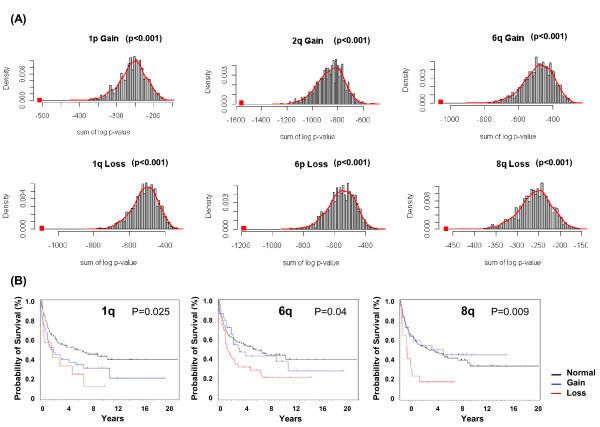
**Association of additional recurrent abnormalities with gene expression and survival time on 190 DLBCLs**. (A) Permutation tests for the association of recurrent abnormalities with the gene expression. The recurrent gains of 1p, 2q and 6q and losses of 1q, 6p and 8q were shown. For each abnormality, the red square showed the test statistic for the real data. The histogram showed the distribution of the test statistic from 1000 permutations. X axis presents the value of test statistics, which is the sum log p-value between the samples that exhibit the recurrent abnormality and those that are wild type for that abnormality. (B) Kaplan-Meier estimates of overall survival of 190 DLBCLs grouped by the abnormalities. The log rank test was used for the p values among the patient groups characterized by the regions of 1q, 6q and 8q.

We further evaluated the significance of a recurrent gain or loss region being associated with the altered gene expression by a permutation test as described in the method section. We performed 1000 permutations for each region and found that in all of the 1000 permutations, the test statistic for the real data was less than the test statistics of the permuted data (p < 0.001, Figure 8A). We also examined the association of those regions with clinical characteristics of the patients. We plotted overall survival (OS) time of the DLBCL patients characterized by those abnormalities, and found that three of those regions are significantly correlated with the survival time of the patient groups: 1q (p = 0.025), 6q (p = 0.04) and 8q (p = 0.009) (Figure 8B). Those associations revealed that the additional recurrent abnormalities identified by vCGH may be functionally important since the genes in those regions have consistently elevated or decreased level of expression and reflect clinical characteristics of DLBCL patients.

Experimental CGH might report false negative CNAs, for example, CGH kits have technical limitations; the optimal cut-off values may vary among samples when calling a "gain" or "loss"; normal cells in stromal or other reactive elements in the tumor microenviroment may contribute to the signal ratio of tumor versus normal. Other than that, one reason that vCGH has identified additional recurrent abnormalities is that, there are other biological mechanisms which exert control of the expression of a group of syntenic genes other than through chromosomal structural changes. For example, epigenetic modifications, such as DNA methylation and histone modifications, may turn on and off genes in DNA independent of the structural changes. It may be important to check the predicted amplified or deleted regions of these tumor samples for epigenetic alterations. Transcriptional units can also be turned on or off as a group of spatially contiguous genes which may resemble, but not due to, chromosomal structural changes. As another example, UniParental Disomy (UPD) occurs when a cell has two copies of a chromosome, or part of a chromosome, from one parent and no copies from the other parent. UPD can result in over- or uder- expression of genes in the affected regions if these genes have undergone genomic imprinting. Therefore, vCGH may identify not only the gain and loss regions caused by chromosomal structural changes, but also the apparent ("gain") or silenced ("loss") regions by other biological mechanisms. Those recurrent abnormalities may also be important to cancer biology and the clinical outcome of the patients. Additionally, with increasing evidence of polymorphic genomic variation in genome it is more important to critically look at structural changes and its influence on gene expression status.

### vCGH prediction on an independent dataset of 176 DLBCLs

**Figure 9:**
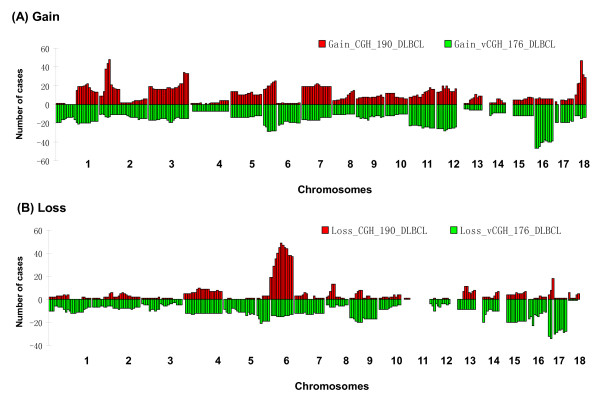
**vCGH prediction on an independent cohort of 176 DLBCLs**. (A) Gain. (B) Loss. The gains or losses identified by CGH for the 190 DLBCLs were shown above the X-axis. The gains or losses predicted by vCGH for the 176 DLBCLs were shown below the X-axis. On X axis, each bar represents a cytoband ordered from pter to qter, from chr1 to chr18. On Y axis, the height of each bar indicates the number of cases horboring a gain or loss on a cytoband.

We applied vCGH which is trained by the paired GEP and CGH data on the 190 DLBCLs, to an independent dataset of 176 DLBCLs with the GEP data [42]. The GEP data of the 176 DLBCLs were downloaded at http://www.broadinstitute.org/cgi-bin/cancer/publications/pub_paper.cgi?mode=view&paper_id=102[42]. Since the CGH data was not available for the 176 DLBCLs, we compared the vCGH-predicted CNAs for the 176 DLBCLs with the CGH-identified CNAs for the 190 DLBCLs because a specific tumor type would feature specific genetic abnormalities even in different patient cohorts. Figure 9 showed the prediction results on the 176 DLBCLs in comparison with the CGH data on the 190 DLBCLs. Since the two patient cohorts are completely independent, we observed some differences in recurrent abnormalities between the two cohorts, especially in losses. However we do observe overall similarity between the two cohorts, such as gains of 1q, 2p14-p16, chr3, chr5, 6p, chr7 and chr9, and losses of chr4, 6q, 13q and 17p. Those recurrent regions have also been reported in another independent aCGH study on 99 DLBCLs [43].

There are some limitations of vCGH due to utilization of transcripts-based GEP data. For example, it may not predict well for regions with few genes (such as "gene desert"), or if the genes in a region are generally not expressed at a sufficiently high level on GEP in even normal status. vCGH is also limited by the design of the GEP arrays. For example, on Affymetrix HG-U133 plus 2 microarrays, there are no probes designed on the p arms of chromosomes 13, 14, 15, 21 and 22. Therefore, vCGH cannot predict gains or losses on those chromosomal regions.

## Conclusions

We proposed a novel computational approach, vCGH, to predict genetic abnormalities from the GEP data in tumors. In addition to the wealth of GEP data already publicly available, vCGH also takes advantage of the paired GEP and CGH data on the same tumor samples in training to infer functionally relevant CNA regions. CNA regions identified by CGH alone in principle define only the chromosomal structural changes; however, the functional effects of CNAs can be reflected by altered gene expression and might be more important to the tumorigenesis. vCGH was constructed on HMMs to capture two primary sources of uncertainty embedded in genomic data: the significant but subtle correlations between GEP and CGH, and the sequential transitions of CNAs along a chromosome. We applied vCGH to two large cohorts of lymphoma samples on which both GEP and CGH experiments were performed, including 190 DLBCLs and 64 MCLs. Using cross-validation, vCGH achieved 80% sensitivity, 90% specificity and 90% accuracy in predicting gains and losses as compared to the experimental CGH on the same tumor samples. In addition to the recurrent gains and losses that are concordant with those by the experimental CGH, vCGH also identified a few recurrent abnormalities not shown by CGH, such as gains of 6q and losses of 1q and 8q on DLBCL, and those regions are significantly correlated with the patients' outcomes. As vCGH utilized both genomic and transcriptomic data, it can identify not only gains and losses by chromosomal structural changes, but also abnormal genomic regions activated or silenced by other mechanisms. We presented the results of vCGH on lymphoma samples, but vCGH is a general computational tool which can be applied to other tumor types and may significantly enhance the identification of functionally important abnormal genomic regions in cancer research.

## Competing interests

The authors declare that they have no competing interests.

## Authors' contributions

HG designed the study, implemented the model, performed the data analysis and drafted the manuscript. JI assisted in GEP and CGH data analysis. WCC contributed to the conceptual development of the model, provided the GEP and CGH data and supervised the study. HAA contributed to the algorithm design and supervised the study. All authors read and approved the final manuscript.

## Pre-publication history

The pre-publication history for this paper can be accessed here:

http://www.biomedcentral.com/1755-8794/4/32/prepub

## Supplementary Material

Additional file 1 **Viterbi, Forward and Backward Algorithms**. Word DOC file. Click here for file

Additional file 2 **Supplemental Tables**. Word DOC containing Supplemental table S1, S2, S3 and S4. Click here for file
