# Supplementary material for: Virtual CGH: an integrative approach to predict genetic abnormalities from gene expression microarray data applied in lymphoma
Source: BMC Med Genomics. 2011 Apr 12;4:32. doi: 10.1186/1755-8794-4-32 (PMC3086850; doi:10.1186/1755-8794-4-32)
Supplement: Additional file 1 — Viterbi, Forward and Backward Algorithms. Word DOC file. [file 1755-8794-4-32-S1.DOC]

**Viterbi, Forward and Backward Algorithms.**

| **Algorithm (1)** Viterbi: |
| --- |
| Initialization (*i*=0): $v_{0}(0)=1\text{, }v_{k}(0)=0\text{ for }k>0.$ Recursion (*i*=1…*L*): $v_{l}(i)=e_{l}(x_{i})\max_{k} (v_{k}(i-1)a_{kl});ptr(l)=\mathop{\mathrm{argmax}}\limits_{k} (v_{k}(i-1)a_{kl}).$ Termination: $P(x,\pi^{*})=\max_{k} (v_{k}(L)a_{k0});\mathop{\pi_{L}^{*}=\mathrm{argmax}}\limits_{k} (v_{k}(L)a_{k0}).$ Traceback (*i*=1…*L*): $\pi_{i-1}^{*}=ptr_{i}(\pi_{i}^{*}).$ |

| **Algorithm (2)** Forward: |
| --- |
| Initialization (*i*=0): $f_{0}(0)=1\text{, }f_{k}(0)=0\text{ for }k>0.$ Recursion (*i*=1…*L*): $f_{l}(i)=e_{l}(x_{i})\sum_{k} f_{k}(i-1)a_{kl}.$ Termination: $P(x)=\sum_{k} f_{k}(L)a_{k0}.$ |

| **Algorithm (3)** Backward: |
| --- |
| Initialization (*i*=L): $b_{k}(L)=a_{k0}\text{ for }k>0.$ Recursion (*i*=*L-*1…1): $b_{k}(i)=\sum_{l} e_{l}(x_{i+1})a_{kl}b_{l}(i+1).$ Termination: $P(x)=\sum_{l} e_{l}(x_{1})a_{0l}b_{l}(1).$ |

*k* and *l* $\in\{H_{+},H_{-},H_{o},L_{+},L_{-},L_{o},M_{+},M_{-},M_{o}\}\text{ }$and *x_i_*$\in\{H,L,M\}$.

Viterbi decoding is a dynamic programming algorithm. Suppose the probability *v_k_*(*i*-1) of the most probable path ending in state *k* with observation *x_i_*_-1_ is known for all the states *k*, then the probability *v_l_*(*i*) corresponding to the observation *x_i_* with the state *l* can be calculated as in Eq. (1). The entire path *π* can be found recursively.

$v_{l}(i)=e_{l}(x_{i})\max_{k} (v_{k}(i-1)a_{kl})$ (1)

where *a_kl_* is the transition probability, *e_l_*(*x_l_*) is the emission probability, *k* and *l* $\in\{H_{+},H_{-},H_{o},L_{+},L_{-},L_{o},M_{+},M_{-},M_{o}\}\text{ }$and *x_i_*$\in\{H,L,M\}$.

Posterior decoding is derived from Forward and Backward algorithms, which are similar dynamic programming procedures to Viterbi by replacing the maximization steps with sums to obtain the full probability for all possible paths. In Forward algorithm, $f_{k}(i)=P(x_{1}\ldots x_{i},\pi_{i}=k)$ is the forward variable, representing the full probability for all the probable paths ending in state *k* with observation up to and including *x_i_*. Then $f_{l}(i+1)$, corresponding to the observation up to and including *x_i_*_+1_ and ending in state *l*, can be calculated by the recursion in Eq.(2). In Backward algorithm, the backward variable $b_{k}(i)=P(x_{i+1}\ldots x_{L}|\pi_{i}=k)$ is analogous to *f_k_*(*i*), but instead obtained by a backward recursion starting at the end of the sequence, as in Eq.(3).

$f_{l}(i+1)=e_{l}(x_{i+1})\sum_{k} f_{k}(i)a_{kl}$ (2)

$b_{k}(i)=\sum_{l} e_{l}(x_{i+1})a_{kl}b_{l}(i+1)$ (3)

where *a_kl_* is the transition probability, *e_l_*(*x_l_*) is the emission probability, *k* and *l* $\in\{H_{+},H_{-},H_{o},L_{+},L_{-},L_{o},M_{+},M_{-},M_{o}\}\text{ }$and *x_i_*$\in\{H,L,M\}$.

Having *f_k_*(*i*) and *b_k_*(*i*), given the emitted sequence *x,* the posterior probability that observation *x_i_* comes from a state *k* is shown in Eq. (4), and that observation *x_i_* comes from all possible states in the specific set is shown in Eq. (5). Then we concatenate the most probable state at each position to form the entire CGH state path.

$P(\pi_{i}=k|x)=f_{k}(i)b_{k}(i)/P(x)$ (4)

$G(i|x)=\sum_{k} P(\pi_{i}=k|x)g(k)$ (5)

where *g*(*k*) is a function defined on the states, *g*(*k*) = 1 for *k*∈{*H*_+_, *L*_+_, *M*_+_}, *g*(*k*) = -1 for *k*∈{*H*-, *L*-, *M*-} and *g*(*k*) = 0 for *k*∈{*H_o_*, *L_o_*, *M_o_*}.
