# Supplementary material for: Virtual CGH: an integrative approach to predict genetic abnormalities from gene expression microarray data applied in lymphoma
Source: BMC Med Genomics. 2011 Apr 12;4:32. doi: 10.1186/1755-8794-4-32 (PMC3086850; doi:10.1186/1755-8794-4-32)
Supplement: Additional file 2 — Supplemental Tables. Word DOC containing Supplemental table S1, S2, S3 and S4. [file 1755-8794-4-32-S2.DOC]

## Supplemental Tables

## Supplemental Table 1. Sensitivity, specificity and accuracy of vCGH on individual chromosomes on DLBCL dataset

| **Gain** | **Sensitivity** (# probesets) | | **Specirifity** (# probesets) | | **Accuracy** (# probesets) | |
| --- | --- | --- | --- | --- | --- | --- |
| chr1 | 67.1% | (22696/33817) | 86.4% | (630981/730121) | 85.6% | (653677/763938) |
| chr2 | 55.8% | (14944/26804) | 88.9% | (454928/511657) | 87.3% | (469872/538461) |
| chr3 | 83.5% | (36154/43315) | 89.6% | (357465/398945) | 89.0% | (393619/442260) |
| chr4 | 18.0% | (547/3045) | 95.6% | (293435/306915) | 94.8% | (293982/309960) |
| chr5 | 71.1% | (15165/21342) | 86.1% | (292644/339837) | 85.2% | (307809/361179) |
| chr6 | 71.6% | (17787/24846) | 87.7% | (335395/382638) | 86.7% | (353182/407484) |
| chr7 | 73.8% | (29292/39698) | 87.0% | (295763/340003) | 85.6% | (325055/379701) |
| chr8 | 68.2% | (8967/13141) | 92.0% | (238227/258830) | 90.9% | (247194/271971) |
| chr9 | 72.5% | (9442/13021) | 93.7% | (268538/286544) | 92.8% | (277980/299565) |
| chr10 | 79.4% | (10732/13513) | 91.4% | (268680/293801) | 90.9% | (279412/307314) |
| chr11 | 66.9% | (17115/25578) | 84.0% | (323871/385686) | 82.9% | (340986/411264) |
| chr12 | 83.5% | (26905/32231) | 85.3% | (305535/358243) | 85.1% | (332440/390474) |
| chr13 | 82.4% | (3860/4684) | 94.5% | (149413/158045) | 94.2% | (153273/162729) |
| chr14 | 72.4% | (3214/4442) | 95.8% | (241796/252409) | 95.4% | (245010/256851) |
| chr15 | 73.6% | (5713/7760) | 90.8% | (214052/235672) | 90.3% | (219765/243432) |
| chr16 | 89.6% | (8849/9879) | 89.9% | (258097/287229) | 89.8% | (266946/297108) |
| chr17 | 53.8% | (4326/8036) | 88.6% | (346727/391510) | 87.9% | (351053/399546) |
| chr18 | 70.2% | (12000/17095) | 91.1% | (106664/117095) | 88.4% | (118664/134190) |
| **Loss** | **Sensitivity** (# probesets) | | **Specirifity** (# probesets) | | **Accuracy** (# probesets) | |
| chr1 | 82.8% | (6958/8406) | 94.3% | (712777/755532) | 94.2% | (719735/763938) |
| chr2 | 86.0% | (6021/7000) | 95.1% | (505295/531461) | 95.0% | (511316/538461) |
| chr3 | 92.2% | (1622/1759) | 97.6% | (429805/440501) | 97.6% | (431427/442260) |
| chr4 | 93.0% | (11295/12145) | 88.9% | (264833/297815) | 89.1% | (276128/309960) |
| chr5 | 97.1% | (1030/1061) | 99.0% | (356482/360118) | 99.0% | (357512/361179) |
| chr6 | 68.7% | (28926/42079) | 88.8% | (324487/365405) | 86.7% | (353413/407484) |
| chr7 | 65.0% | (2433/3744) | 95.7% | (359709/375957) | 95.4% | (362142/379701) |
| chr8 | 45.0% | (2168/4819) | 92.2% | (246338/267152) | 91.4% | (248506/271971) |
| chr9 | 49.1% | (1830/3730) | 94.2% | (278560/295835) | 93.6% | (280390/299565) |
| chr10 | 59.3% | (2360/3980) | 94.3% | (286081/303334) | 93.9% | (288441/307314) |
| chr11 | 0% | (0/178) | 100% | (411086/411086) | 100% | (411086/411264) |
| chr12 | 43.6% | (312/716) | 99.2% | (386504/389758) | 99.1% | (386816/390474) |
| chr13 | 65.4% | (3431/5244) | 94.5% | (148847/157485) | 93.6% | (152278/162729) |
| chr14 | 88.7% | (4152/4682) | 95.3% | (240337/252169) | 95.2% | (244489/256851) |
| chr15 | 73.6% | (5015/6817) | 92.2% | (218270/236615) | 91.7% | (223285/243432) |
| chr16 | 58.1% | (769/1324) | 97.8% | (289331/295784) | 97.6% | (290100/297108) |
| chr17 | 77.1% | (7334/9508) | 94.8% | (369758/390038) | 94.4% | (377092/399546) |
| chr18 | 66.3% | (1329/2004) | 98.4% | (130005/132186) | 97.9% | (131334/134190) |

## Supplemental Table 2. Sensitivity, specificity and accuracy of vCGH on individual chromosomes on MCL dataset

| **Gain** | **Sensitivity** (# probesets) | | **Specirifity** (# probesets) | | **Accuracy** (# probesets) | |
| --- | --- | --- | --- | --- | --- | --- |
| chr1 | 0% | (0/314) | 99.7% | (306952/307846) | 99.6% | (306952/308160) |
| chr2 | 87.5% | (2641/3018) | 94.3% | (204621/217014) | 94.2% | (207262/220032) |
| chr3 | 55.4% | (13011/23482) | 79.1% | (125322/158534) | 76.0% | (138333/182016) |
| chr4 | NA | (0/0) | 100% | (128192/128192) | 100% | (128192/128192) |
| chr5 | NA | (0/0) | 100% | (147200/147200) | 100% | (147200/147200) |
| chr6 | 0% | (0/668) | 98.9% | (163019/164836) | 98.5% | (163019/165504) |
| chr7 | 91.9% | (7220/7854) | 86.4% | (126985/147026) | 86.7% | (134205/154880) |
| chr8 | 70.6% | (4441/6286) | 89.7% | (96118/107122) | 88.7% | (100559/113408) |
| chr9 | 0% | (0/425) | 100% | (120343/120343) | 99.6% | (120343/120768) |
| chr10 | 0% | (0/487) | 100% | (127129/127129) | 99.6% | (127129/127616) |
| chr11 | 55.7% | (1109/1991) | 92.3% | (152309/165049) | 91.8% | (153418/167040) |
| chr12 | 96.6% | (5213/5394) | 89.8% | (138253/153966) | 90.0% | (143466/159360) |
| chr13 | 0% | (0/121) | 100% | (70151/70151) | 99.8% | (70151/70272) |
| chr14 | 58.6% | (374/638) | 97.8% | (100250/102466) | 97.6% | (100624/103104) |
| chr15 | 52.2% | (2362/4525) | 90.4% | (86873/96147) | 88.6% | (89235/100672) |
| chr16 | 88.7% | (2636/2973) | 93.3% | (108543/116387) | 93.1% | (111179/119360) |
| chr17 | 61.9% | (1271/2052) | 93.5% | (147127/157436) | 93.0% | (148398/159488) |
| chr18 | 67.1% | (3257/4856) | 90.3% | (46544/51528) | 88.3% | (49801/56384) |
| **Loss** | **Sensitivity** (# probesets) | | **Specirifity** (# probesets) | | **Accuracy** (# probesets) | |
| chr1 | 35.2% | (5461/15528) | 86.1% | (252015/292632) | 83.6% | (257476/308160) |
| chr2 | 58.6% | (588/1004) | 98.8% | (216391/219028) | 98.6% | (216979/220032) |
| chr3 | 42.4% | (1191/2811) | 97.5% | (174680/179205) | 96.6% | (175871/182016) |
| chr4 | 0% | (0/450) | 100% | (127742/127742) | 99.6% | (127742/128192) |
| chr5 | 0% | (0/403) | 100% | (146797/146797) | 99.7% | (146797/147200) |
| chr6 | 70.1% | (8945/12769) | 85.5% | (130524/152735) | 84.3% | (139469/165504) |
| chr7 | 68.7% | (1166/1697) | 96.1% | (147233/153183) | 95.8% | (148399/154880) |
| chr8 | 70.8% | (2821/3986) | 91.2% | (99762/109422) | 90.5% | (102583/113408) |
| chr9 | 66.5% | (10688/16076) | 87.5% | (91622/104692) | 84.7% | (102310/120768) |
| chr10 | 52.7% | (648/1229) | 96.8% | (122389/126387) | 96.4% | (123037/127616) |
| chr11 | 33.7% | (2477/7357) | 86.4% | (137984/159683) | 84.1% | (140461/167040) |
| chr12 | 100% | (1097/1097) | 97.5% | (154295/158263) | 97.5% | (155392/159360) |
| chr13 | 77.3% | (11196/14493) | 86.1% | (48038/55779) | 84.3% | (59234/70272) |
| chr14 | 71.5% | (1081/1512) | 97.6% | (99177/101592) | 97.2% | (100258/103104) |
| chr15 | 0% | (0/223) | 100% | (100449/100449) | 99.8% | (100449/100672) |
| chr16 | 100% | (991/991) | 97.6% | (115532/118369) | 97.6% | (116523/119360) |
| chr17 | 79.1% | (3246/4104) | 93.1% | (144696/155384) | 92.8% | (147942/159488) |
| chr18 | 21.6% | (66/306) | 99.6% | (55831/56078) | 99.1% | (55897/56384) |

## Supplemental Table 3. Comparison of sensitivity, specificity and accuracy of vCGH on gene level (vCGH) and on cytoband level (c_vCGH) on individual chromosomes on DLBCL dataset

|  | **Sensitivity** | | **Specificity** | | **Accuracy** |  |
| --- | --- | --- | --- | --- | --- | --- |
| **Gain** | **vCGH** | **c_vCGH** | **vCGH** | **c_vCGH** | **vCGH** | **c_vCGH** |
| chr1 | 67.1% | 69.0% | 86.4% | 84.7% | 85.6% | 83.9% |
| chr2 | 55.8% | 57.1% | 88.9% | 88.6% | 87.3% | 87.0% |
| chr3 | 83.5% | 83.5% | 89.6% | 88.8% | 89.0% | 88.3% |
| chr4 | 18.0% | 23.1% | 95.6% | 95.7% | 94.8% | 95.0% |
| chr5 | 71.1% | 75.4% | 86.1% | 85.6% | 85.2% | 85.0% |
| chr6 | 71.6% | 67.9% | 87.7% | 87.5% | 86.7% | 86.7% |
| chr7 | 73.8% | 73.6% | 87.0% | 87.0% | 85.6% | 85.6% |
| chr8 | 68.2% | 71.3% | 92.0% | 91.4% | 90.9% | 90.6% |
| chr9 | 72.5% | 83.2% | 93.7% | 92.6% | 92.8% | 92.2% |
| chr10 | 79.4% | 80.7% | 91.4% | 90.6% | 90.9% | 90.1% |
| chr11 | 66.9% | 68.7% | 84.0% | 83.6% | 82.9% | 82.7% |
| chr12 | 83.5% | 85.8% | 85.3% | 84.9% | 85.1% | 85.0% |
| chr13 | 82.4% | 78.7% | 94.5% | 93.9% | 94.2% | 93.4% |
| chr14 | 72.4% | 82.1% | 95.8% | 95.6% | 95.4% | 95.4% |
| chr15 | 73.6% | 70.3% | 90.8% | 90.3% | 90.3% | 89.7% |
| chr16 | 89.6% | 95.1% | 89.9% | 89.8% | 89.8% | 90.0% |
| chr17 | 53.8% | 56.4% | 88.6% | 88.2% | 87.9% | 87.6% |
| chr18 | 70.2% | 72.2% | 91.1% | 90.8% | 88.4% | 88.4% |
| **Loss** | **vCGH** | **c_vCGH** | **vCGH** | **c_vCGH** | **vCGH** | **c_vCGH** |
| chr1 | 82.8% | 92.1% | 94.3% | 93.2% | 94.2% | 93.2% |
| chr2 | 86.0% | 86.4% | 95.1% | 94.8% | 95.0% | 94.7% |
| chr3 | 92.2% | 88.2% | 97.6% | 97.1% | 97.6% | 97.1% |
| chr4 | 93.0% | 92.1% | 88.9% | 89.0% | 89.1% | 89.1% |
| chr5 | 97.1% | 85.7% | 99.0% | 98.5% | 99.0% | 98.4% |
| chr6 | 68.7% | 65.7% | 88.8% | 89.3% | 86.7% | 86.2% |
| chr7 | 65.0% | 65.7% | 95.7% | 95.6% | 95.4% | 95.2% |
| chr8 | 45.0% | 43.5% | 92.2% | 91.9% | 91.4% | 90.9% |
| chr9 | 49.1% | 44.2% | 94.2% | 94.1% | 93.6% | 93.3% |
| chr10 | 59.3% | 55.6% | 94.3% | 93.6% | 93.9% | 93.1% |
| chr11 | 0.0% | 0.0% | 100.0% | 100.0% | 100.0% | 99.9% |
| chr12 | 43.6% | 75.0% | 99.2% | 99.2% | 99.1% | 99.1% |
| chr13 | 65.4% | 60.0% | 94.5% | 94.1% | 93.6% | 92.9% |
| chr14 | 88.7% | 96.2% | 95.3% | 95.0% | 95.2% | 95.0% |
| chr15 | 73.6% | 74.1% | 92.2% | 91.5% | 91.7% | 91.1% |
| chr16 | 58.1% | 58.3% | 97.8% | 96.9% | 97.6% | 96.6% |
| chr17 | 77.1% | 83.8% | 94.8% | 93.9% | 94.4% | 93.7% |
| chr18 | 66.3% | 61.1% | 98.4% | 98.2% | 97.9% | 97.6% |

## Supplemental Table 4. Comparison of sensitivity, specificity and accuracy of vCGH on gene level (vCGH) and on cytoband level (c_vCGH) on individual chromosomes on MCL dataset

|  | **Sensitivity** | | **Specificity** | | **Accuracy** |  |
| --- | --- | --- | --- | --- | --- | --- |
| **Gain** | **vCGH** | **c_vCGH** | **vCGH** | **c_vCGH** | **vCGH** | **c_vCGH** |
| chr1 | 0.0% | 0.0% | 99.7% | 99.5% | 99.6% | 99.4% |
| chr2 | 87.5% | 86.4% | 94.3% | 94.2% | 94.2% | 94.1% |
| chr3 | 55.4% | 56.8% | 79.1% | 78.2% | 76.0% | 75.5% |
| chr4 | NA | NA | 100.0% | 100.0% | 100.0% | 100.0% |
| chr5 | NA | NA | 100.0% | 100.0% | 100.0% | 100.0% |
| chr6 | 0.0% | 0.0% | 98.9% | 98.5% | 98.5% | 98.0% |
| chr7 | 91.9% | 92.5% | 86.4% | 85.8% | 86.7% | 86.1% |
| chr8 | 70.6% | 88.2% | 89.7% | 89.2% | 88.7% | 89.2% |
| chr9 | 0.0% | 0.0% | 100.0% | 100.0% | 99.6% | 99.9% |
| chr10 | 0.0% | 0.0% | 100.0% | 100.0% | 99.6% | 99.6% |
| chr11 | 55.7% | 70.0% | 92.3% | 91.8% | 91.8% | 91.5% |
| chr12 | 96.6% | 89.3% | 89.8% | 89.6% | 90.0% | 89.6% |
| chr13 | 0.0% | 0.0% | 100.0% | 100.0% | 99.8% | 99.7% |
| chr14 | 58.6% | 66.7% | 97.8% | 97.2% | 97.6% | 97.0% |
| chr15 | 52.2% | 50.0% | 90.4% | 90.4% | 88.6% | 88.8% |
| chr16 | 88.7% | 88.9% | 93.3% | 92.1% | 93.1% | 92.0% |
| chr17 | 61.9% | 66.7% | 93.5% | 93.0% | 93.0% | 92.7% |
| chr18 | 67.1% | 70.6% | 90.3% | 89.1% | 88.3% | 87.5% |
| **Loss** | **vCGH** | **c_vCGH** | **vCGH** | **c_vCGH** | **vCGH** | **c_vCGH** |
| chr1 | 35.2% | 35.4% | 86.1% | 85.5% | 83.6% | 82.7% |
| chr2 | 58.6% | 71.4% | 98.8% | 98.5% | 98.6% | 98.4% |
| chr3 | 42.4% | 38.9% | 97.5% | 96.8% | 96.6% | 96.1% |
| chr4 | 0.0% | 0.0% | 100.0% | 100.0% | 99.6% | 99.8% |
| chr5 | 0.0% | 0.0% | 100.0% | 100.0% | 99.7% | 99.9% |
| chr6 | 70.1% | 70.6% | 85.5% | 85.2% | 84.3% | 84.0% |
| chr7 | 68.7% | 77.8% | 96.1% | 94.8% | 95.8% | 94.6% |
| chr8 | 70.8% | 77.8% | 91.2% | 90.4% | 90.5% | 90.0% |
| chr9 | 66.5% | 70.9% | 87.5% | 84.9% | 84.7% | 83.2% |
| chr10 | 52.7% | 44.4% | 96.8% | 96.2% | 96.4% | 95.6% |
| chr11 | 33.7% | 31.7% | 86.4% | 86.1% | 84.1% | 83.6% |
| chr12 | 100.0% | 100.0% | 97.5% | 97.0% | 97.5% | 97.0% |
| chr13 | 77.3% | 77.0% | 86.1% | 84.8% | 84.3% | 83.1% |
| chr14 | 71.5% | 100.0% | 97.6% | 97.7% | 97.2% | 97.7% |
| chr15 | 0.0% | 0.0% | 100.0% | 100.0% | 99.8% | 99.9% |
| chr16 | 100.0% | 100.0% | 97.6% | 96.5% | 97.6% | 96.6% |
| chr17 | 79.1% | 83.3% | 93.1% | 91.7% | 92.8% | 91.6% |
| chr18 | 21.6% | 33.3% | 99.6% | 99.5% | 99.1% | 99.0% |
